# P-874. Clinical Characteristics and Outcomes Associated with Enterococcal Bloodstream Infections at an Integrated Healthcare Network

**DOI:** 10.1093/ofid/ofae631.1065

**Published:** 2025-01-29

**Authors:** Ryan Rothman, G E O R G E BCHECH, Saleh Saleh, Nitin Bhanot, Rasha Abdulmassih

**Affiliations:** Allegheny General Hospital/Allegheny Health Network, Pittsburgh, Pennsylvania; Allegheny General Hospital/Allegheny Health Network, Pittsburgh, Pennsylvania; Allegheny Health Network, Pittsburgh, Pennsylvania; Allegheny Health Network, Pittsburgh, Pennsylvania; AHN, pittsburgh, Pennsylvania

## Abstract

**Background:**

*Enterococcus* spp. represent a frequent cause of blood stream infections in the United States. We wanted to gather data on clinical characteristics and outcomes associated with enterococcal bloodstream infections encountered at our quaternary care integrated health network.

**Table 1:**
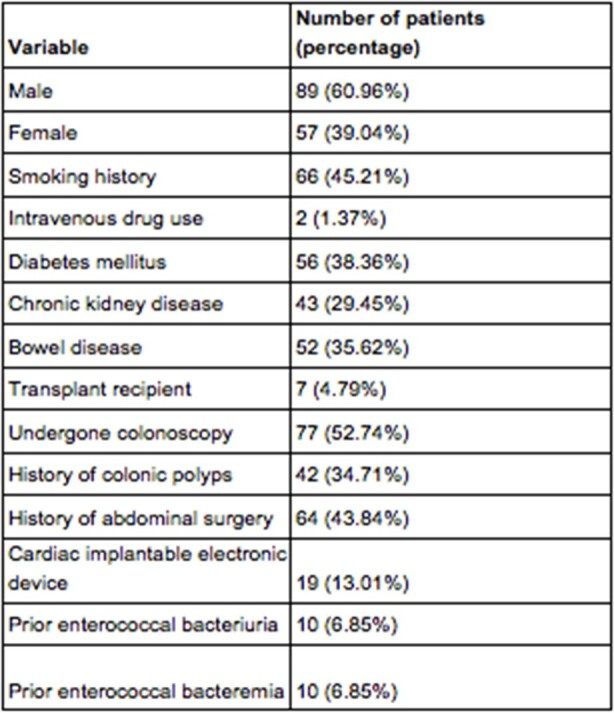
Patient demographics and comorbidities Baseline characteristics of patients reviewed in the study, including their demographic data and underlying medical history

**Methods:**

We conducted a multicenter retrospective chart review from 2022 to 2023 of patients hospitalized (community or healthcare acquired) with enterococcal bacteremia. Information on demographics, comorbidities, duration of treatment, readmission rates, and mortality were obtained.

**Figure 1:**
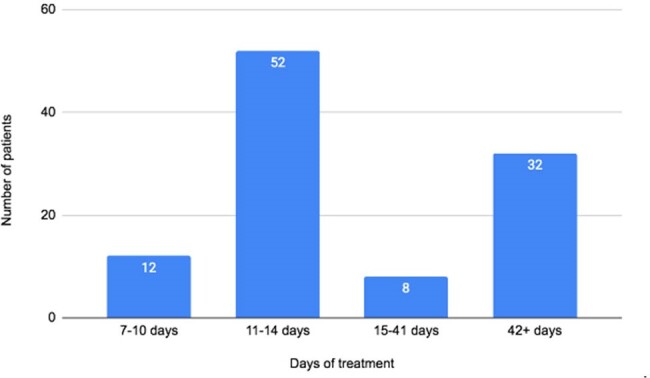
Prescribed treatmet duration

Treatment duration in patients who completed their prescribed antimicrobial therapy course

**Results:**

146 patients (mean age 67.5 years) were identified with enterococcal bacteremia. Demographics and comorbidities are shown in **Table 1**. Prescribed treatment duration varied based on the underlying clinical presentation, and is outlined in **Figure 1**, which only includes patients who were able to complete the prescribed course. 30-day all-cause readmission was 34/146 (23.3%), with only 1 readmission due to recurrence of bacteremia. 30-day all-cause mortality was 51/146 (35%). Most of these patients (48/51; 94%) died prior to completing the antimicrobial therapy; 3/48 (6%) patients died following completion of an 11-14 day treatment course.

**Conclusion:**

There was a significant burden of enterococcal bacteremia at our institution during the study period. Although 30-day readmission was seen in about 23% of patients, there was only 1 patient who was readmitted for recurrent bacteremia, notably in a patient who had not yet finished his prescribed course of antibiotics. Additionally, we encountered a high all-cause mortality rate of 35%, with the majority dying while on active antimicrobial therapy. These findings are congruent with other studies measuring the mortality rate of enterococcal bacteremia. This study highlights the importance of identifying high-risk comorbidities in patients that might portend a poor prognosis, and helps to educate both providers and patients on the overall prognosis of enterococcal bacteremia.

**Disclosures:**

**All Authors**: No reported disclosures

